# ST segment elevation associated with hydrochloric acid ingestion: A case report

**DOI:** 10.1097/MD.0000000000008819

**Published:** 2017-11-27

**Authors:** I-Jeng Yeh, Kuan-Ting Liu

**Affiliations:** a Department of Emergency Medicine, Kaohsiung Medical University Hospital; b Institute of Clinical Medicine, College of Medicine; c School of Medicine, College of Medicine, Kaohsiung Medical University, Kaohsiung, Taiwan.

**Keywords:** electrocardiography, hydrochloric acid, intoxication, ST elevation myocardial infarction

## Abstract

**Rationale:**

Electrocardiography (ECG) was used to diagnose acute coronary syndrome, but many other diseases may also result in ST segment change. We report one case of ingested hydrochloric acid present with ST segment elevation in the ECG. However, subsequent coronary angiography did not reveal significant coronary occlusion.

**Patient concerns:**

An 83-year-old female was transferred to our emergency department (ED) from the branch hospital due to ingestion of toilet bowl cleaner containing 9.5% hydrochloric acid. She complained about chest pain and 12-lead ECG showed ST segment elevation at lead II, III, and aVF. The blood examinations revealed elevation of aspartate transaminase (69 IU/L), thrombocytopenia (62,000/μL), and acidosis (pH 7.311, pCO_2_ 27 mm Hg, HCO_3_ 13.3 mmol/L). Creatine kinase-MB and troponin I did not elevate then.

**Diagnoses:**

After transferred to our ED, coronary angiography was done within 1 hour. Angiography showed 60% stenosis in the segment 7 of left anterior descending coronary artery and 30% nonsignificant stenosis in the segment 2 of right coronary artery, with no apical ballooning. No significant lesion consistent with ST segment elevation myocardial infarction was found.

**Interventions:**

Conservative treatment was chosen.

**Outcomes:**

Bradycardia was followed by cardiac arrest that developed 4 hours later. Cardiopulmonary resuscitation was applied and the patient became shock status after return of spontaneous circulation. The patient was admitted to the intensive care unit and expired on next day.

**Lessons:**

Patients of ingested hydrochloric acid present with ST segment elevation in the ECG may not indicate coronary artery disease. This ECG finding may be a poor prognostic index in such patients.

## 1 Introduction

Electrocardiography (ECG) was used to diagnose acute coronary syndrome, but many other diseases may also result in ST segment change in the ECG. It has been reported that hydrochloric acid ingestion may result in ST segment elevation myocardial infarction (STEMI).^[1–2]^ We report one case of ingested hydrochloric acid present with ST segment elevation in the ECG. However, subsequent coronary angiography did not reveal significant coronary occlusion.

The ethical approval was not necessary for this case report article under the regulations of institutional review board of the Kaohsiung Medical University Hospital.

## 2 Case report

**Figure 1:**
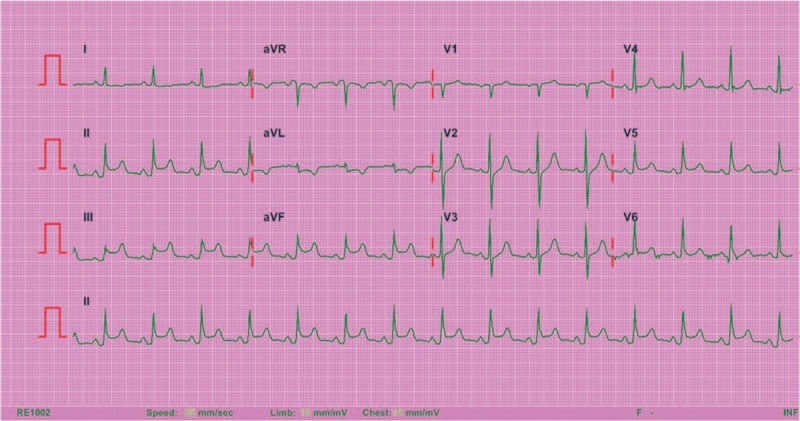
Electrocardiogram of the patient showing ST-segment elevations in leads II, III, and aVF, which is compatible with acute inferior myocardial infarction.

An 83-year-old female was transferred to our emergency department (ED) from the branch hospital due to hydrochloric acid ingestion about one and a half hours previously. She was a case of major depressive disorder. She drank toilet bowl cleaner containing 9.5% hydrochloric acid. Subsequently, she was sent to our branch hospital ED. She complained about chest pain and 12-lead ECG was done. ST segment elevation was noted at lead II, III, and aVF (Fig. 1). The blood examination included complete blood count, glucose, aspartate transaminase, alanine transaminase, blood urea nitrogen, creatinine, sodium, potassium, creatine kinase, creatine kinase-MB, Troponin I, arterial blood gas, prothrombin time, and partial thromboplastin time. Elevated aspartate transaminase (69 IU/L), thrombocytopenia (62000/μL), and acidosis (pH 7.311, pCO_2_ 27 mm Hg, HCO_3_ 13.3 mmol/L) were noted. Cardiac enzyme test did not elevate then (creatine kinase 36 IU/L, creatine kinase-MB 1.8 ng/mL, troponin I 0.02 ng/mL).

Under the impression of STEMI, she was transferred to our ED within 1 hour. Coronary angiography was done within 1 hour after arrival. Angiography showed 60% stenosis in the segment 7 of left anterior descending coronary artery and 30% nonsignificant stenosis in the segment 2 of right coronary artery, with no apical ballooning. No significant lesion consistent with STEMI was found. The following venous blood gas showed rapid progress metabolic acidosis (pH 7.066, HCO_3_ 7.9 mm Hg). Bradycardia was followed by cardiac arrest that developed 4 hours later. Cardiopulmonary resuscitation was applied and the patient became shock status after return of spontaneous circulation. Computed tomograpy did not reveal pneumomediastinum but showed diffuse stomach wall thickening and adjacent fluid accumulation. Family decided on conservative treatment and the patient was admitted to the intensive care unit and expired on next day.

## 3 Discussion

This patient was sent to our ED because of hydrochloric acid ingestion. As ECG revealed ST segment elevation over lead II, III, and aVF, emergent coronary angiography was performed under the impression of STEMI. However, no significant lesion consistent with STEMI was found. Previous literature has reported that hydrochloric acid may result in acute myocardial infarction,^[1–2]^ but coronary angiography was not performed in their cases. Coronary angiography was performed in our patient and the examination finding suggested that the ST segment elevation might have been due to mechanisms other than coronary artery occlusion.

Many other esophagus and stomach diseases have been reported as associated with ST segment elevation in ECG, such as distended stomach conduit and expansion of colonic tube used for esophageal reconstruction.^[3–5]^ Heart stimulation or change of anatomy may contribute to the change. Islamoglu had reported ST segment elevation in a patient with hydrogen peroxide ingestion.^[6]^ Coronary angiography was performed, but no thrombotic occlusion was found in coronary artery. They suspected coronary spasm may result in the ECG finding.

Hydrochloric acid ingestion is mainly harmful to the gastrointestinal system, although it may also cause metabolic acidosis, hemolysis, renal failure, and fatality as well. ST segment elevation may also be observed in a few cases, but may not indicate coronary artery disease. As all hydrochloric acid ingested patients with ST elevation in our and previous case reports died,^[1–2]^ this ECG finding may be a poor prognostic index in such patients. We might be advised to consider other examinations other than emergent coronary angiography for patients having ingested hydrochloric acid presenting with ECG abnormality.

## References

[1] Sari I Zengin S Pehlivan Y Fatal myocardial infarction after hydrochloric acid ingestion in a suicide attempt. Am J Emerg Med 2008;26: 634.e5-7. 10.1016/j.ajem.2007.10.009 18534312

[2] Yanturali S Aksay E Atilla R Acute myocardial infarction after hydrochloric acid ingestion. Mt Sinai J Med 2005;72:409–12. 16358168

[3] Asada S Kawasaki T Taniguchi T A case of ST-segment elevation provoked by distended stomach conduit. Int J Cardiol 2006;109:411–3. 15979741 10.1016/j.ijcard.2005.05.036

[4] Chen TY Lin CH Tsai SH Pseudomyocardial infarction caused by expansion of colonic tube used for esophageal reconstruction. Am J Med Sci 2012;344:499–500. 22874620 10.1097/MAJ.0b013e318259b86b

[5] Kaneko Y Nakajima T Irie T Brugada-type ST-elevation associated with writhing of a reconstructed esophagus. Intern Med 2013;52:2287–8. 24088770 10.2169/internalmedicine.52.0795

[6] Islamoglu Y Cil H Atilgan Z Myocardial infarction secondary to unintentional ingestion of hydrogen peroxide. Cardiol J 2012;19:86–8. 22298174 10.5603/cj.2012.0014

